# Developing Implementation Strategies to Support the Uptake of a Risk Tool to Aid Physicians in the Clinical Management of Patients With Syncope: Systematic Theoretical and User-Centered Design Approach

**DOI:** 10.2196/44089

**Published:** 2023-06-13

**Authors:** Geneviève Rouleau, Venkatesh Thiruganasambandamoorthy, Kelly Wu, Bahareh Ghaedi, Phuong Anh Nguyen, Laura Desveaux

**Affiliations:** 1 Institute for Health System Solutions and Virtual Care Women’s College Hospital Toronto, ON Canada; 2 Nursing Department, Université du Québec en Outaouais Saint-Jérôme, QC Canada; 3 Department of Emergency Medicine, University of Ottawa Ottawa, ON Canada; 4 Ottawa Hospital Research Institute, Ottawa Hospital Ottawa, ON Canada; 5 Institute for Better Health, Trillium Health Partners Mississauga, ON Canada; 6 Women's College Research Institute Women's College Hospital Toronto, ON Canada; 7 Institute of Health Policy, Management and Evaluation University of Toronto Toronto, ON Canada

**Keywords:** emergency medicine, physicians, qualitative research, risk management, syncope, user-centered design

## Abstract

**Background:**

The Canadian Syncope Risk Score (CSRS) was developed to improve syncope management in emergency department settings. Evidence-based tools often fail to have the intended impact because of suboptimal uptake or poor implementation.

**Objective:**

In this paper, we aimed to describe the process of developing evidence-based implementation strategies to support the deployment and use of the CSRS in real-world emergency department settings to improve syncope management among physicians.

**Methods:**

We followed a systematic approach for intervention development, including identifying who needs to do what differently, identifying the barriers and enablers to be addressed, and identifying the intervention components and modes of delivery to overcome the identified barriers. We used the Behaviour Change Wheel to guide the selection of implementation strategies. We engaged CSRS end users (ie, emergency medicine physicians) in a user-centered design approach to generate and refine strategies. This was achieved over a series of 3 qualitative user-centered design workshops lasting 90 minutes each with 3 groups of emergency medicine physicians.

**Results:**

A total of 14 physicians participated in the workshops. The themes were organized according to the following intervention development steps: theme 1—identifying and refining barriers and theme 2—identifying the intervention components and modes of delivery. Theme 2 was subdivided into two subthemes: (1) generating high-level strategies and developing strategies prototypes and (2) refining and testing strategies. The main strategies identified to overcome barriers included education in the format of meetings, videos, journal clubs, and posters (to address uncertainty around when and how to apply the CSRS); the development of a web-based calculator and integration into the electronic medical record (to address uncertainty in how to apply the CSRS); a local champion (to address the lack of team buy-in); and the dissemination of evidence summaries and feedback through email communications (to address a lack of evidence about impact).

**Conclusions:**

The ability of the CSRS to effectively improve patient safety and syncope management relies on broad buy-in and uptake across physicians. To ensure that the CSRS is well positioned for impact, a comprehensive suite of strategies was identified to address known barriers.

## Introduction

### Background

Syncope is a prevalent and high-cost problem in emergency departments (EDs) defined as a sudden transient loss of consciousness followed by spontaneous complete recovery [1-4]. It accounted for 15,476,451 ED visits in the United States from 2005 to 2015 [5]. In Canada, there are approximately 160,000 ED visits annually that translate to direct hospital costs of approximately CAD $130 million (approximately US $101 million) [6,7]. Although the cause is often benign, approximately 10% of patients will have serious underlying conditions identified within 30 days [8,9]. Up to half of these conditions will be identified after the initial decision to discharge or admit the patient from the ED, emphasizing the need for supports to improve risk stratification and decision-making in the ED [1,8-10].

The Canadian Syncope Risk Score (CSRS) is a validated risk stratification tool to optimize the accuracy of ED decisions and inform evidence-based clinical actions [9,11]. The tool encompasses the calculation of a risk score from which the following evidence-based practice recommendations for ED disposition are derived: immediate discharge of low-risk patients without subjecting them to further unnecessary testing, consideration for a short course of hospitalization for high-risk patients, and discharge of medium-risk patients with cardiac monitoring and clear information regarding the serious outcome risk. Similar to many decision aids, the uptake of the CSRS is likely to prevent unnecessary hospitalization and improve outcomes for those with underlying conditions through the implementation of a standardized and evidence-based approach to ED syncope management. However, the existence of the CSRS is only part of the solution—physicians must see the value and use the tool in practice to realize the expected benefits.

Health services research shows that many evidence-based practices or interventions fail to demonstrate the anticipated impact as they were not properly implemented, precluding them from achieving a positive impact on clinical practice and patient outcomes [12,13]. Implementation failure might be because of an insufficient understanding of the context in which the implementation process occurs [14,15] or an absence of barriers identification influencing the use of such interventions [16]. The field of implementation science provides systematic approaches and strategies to address the research to practice gap by systematically assessing likely barriers to uptake and identifying implementation strategies to target these barriers [13,17-19].

### Objective

The objective of this developmental work was to describe the process of developing a set of implementation strategies to support the use of the CSRS in real-world ED settings to improve syncope management among physicians. This systematic approach not only identifies which strategies are the most appropriate for the target context but also how they should be implemented and operationalized to mitigate the risk of suboptimal implementation or poor uptake among target users [20].

## Methods

We followed the process for intervention development outlined by French et al [21], including the first three steps: (1) identifying who needs to do what differently, (2) identifying the barriers and enablers to be addressed, and (3) identifying the intervention components and modes of delivery to overcome the identified barriers.

### Study Design

#### Steps 1 and 2: Identifying Who Needs to Do What Differently and the Barriers and Enablers to Be Addressed

We previously identified that emergency medicine physicians need to change their approach to the assessment of syncope, which would influence their subsequent management among patients. The initial qualitative work identified barriers among 41 physicians across 12 Canadian ED sites to both CSRS use and the adoption of its evidence-based practice recommendations [22]. The most salient barriers identified were workflow issues, concerns about continuity of care, the lack of confidence in the CSRS, and the lack of knowledge and skills around how to interpret and apply the CSRS-related criteria for various patient profiles [22]. The most reported enablers were as follows: legitimacy in the decision rule (CSRS), the evidence of safety and benefit to send the patient home, cardiologists and emergency medicine physicians buy-in, and adequate time with the patient.

The understanding of these barriers and enablers was refined and contextualized with other groups of emergency medicine physicians who participated in workshops as part of a user-centered design (UCD) approach, which is presented in step 3. Essentially, the list of barriers was presented to physicians, and they were asked to react out loud to the following questions: Does this list look complete? Are there other barriers you would like to bring in our attention? and What do you think?

#### Step 3: Identifying the Intervention Components and Modes of Delivery to Overcome the Identified Barriers

This step was informed by the Behaviour Change Wheel [23], in which we systematically mapped the barriers identified in qualitative work [22] to theoretical determinants, as presented in the Theoretical Domains Framework (TDF) [24] and the Capability, Opportunity, Motivation, and Behavior (COM-B) model [23]. We went through the qualitative findings and looked at physicians-related barriers and facilitators (eg, the lack of knowledge around the eligibility criteria of using CSRS) and matched them with the corresponding theoretical determinant (“knowledge” [TDF] and “capability” [COM-B]). TDF offers a comprehensive lens to look at cognitive, affective, social, and environmental factors [24] that can influence CSRS uptake, whereas COM-B allows for broader categories of determinants.

Once this granular and systematic theoretical understanding of determinants has been completed, we linked them to evidence-based behavior change techniques (BCTs) [25,26], which are the active ingredients or components of an intervention. This step allows to select the most likely techniques to produce the desired change [23,26,27]. From the literature [23,25,26,28], we identified the most effective BCTs that can address each of the behavioral determinants [23,27]. We defined each BCT, how it addressed determinants, and how we could operationalize them. We assessed the feasibility of using these BCTs through peer debriefing (GR, LD, and Marlena Dang Nguyen) and by using the Acceptability, Practicability, Effectiveness, Affordability, Side-effects, Equity criteria [28]. An excerpt of the process of identifying the intervention components and modes of delivery to overcome the identified barriers is presented in Multimedia Appendix 1 [23,25-30]. Selecting those intervention components (also named “implementation strategies”) was the theoretical groundwork that fed the subsequent steps of our developmental process.

We used a UCD approach to validate and refine our understanding of the barriers and enablers and to identify modes of delivery and operationalization. We engaged emergency medicine physicians who were the end users of the CSRS under a collaborative, participatory, and cocreative lens to pursue the parallel goals of maximizing usability in the context of those targeted by the implementation endeavor and tailoring strategies to users’ local contexts while retaining the core components responsible for their effectiveness [18]. This was achieved over a series of 3 qualitative UCD workshops lasting 90 minutes each with 3 groups of emergency medicine physicians. Data collection was performed on the web through synchronous interactions using the Zoom videoconferencing platform (Zoom Video Communications Inc). The workshop-related processes and content are summarized in detail in Textbox 1. Examples of probing questions in the workshop facilitation guide are presented in Textbox 2. GR facilitated the workshops. She did not know the participants before the study. The facilitator (GR) encouraged a “think-aloud” approach to provide insight into participants’ thought processes and gather feedback on which implementation strategies might be useful and why (or why not). Specifically, participants were asked to share their reactions, sentiments, and thought processes in real time. The goal was not to achieve data saturation.

We followed the COREQ (Consolidated Criteria for Reporting Qualitative Studies) [31] to report the qualitative process (Multimedia Appendix 2).

**Workshops-related processes and content.**

**Workshop 1**

- Material sent: study information, link to complete web-based survey, and 2 scientific papers that support the development and validation of the Canadian Syncope Risk Score (CSRS [9,11])
- Objectives: (1) solicit feedback on previously identified barriers to uptake the CSRS, (2) rank barriers in terms of priority for attention with the Zoom polling function, and (3) discuss and brainstorm which strategies might effectively address the barriers and improve the uptake of the CSRS.
- Analysis: review notes, review audio recording to summarize perceptions and key insights, debrief with team.
- Outcome: create a mock poster in response to participant feedback for discussion at workshop 2.

**Workshop 2**

- Preparatory work: view existing educational videos (n=4) about how CSRS was developed and validated and what are the underlying practice-based recommendations
- Material sent: study information, link to complete web-based survey, link to access the educational videos, and 2 scientific papers that support the development and validation of the CSRS [9,11]
- Objectives: (1) solicit feedback on previously identified barriers to uptake the CSRS (workshop 1), (2) solicit feedback on previously identified strategies (theoretical work), and (3) define parameters of operationalization of the strategies (eg, mode of delivery, materials, and content)
- Analysis: review notes, review audio recording to summarize perceptions and key insights, debrief with team, participants’ comments on the workshop summary
- Outcome: refine the mock poster.

**Workshop 3**

- Preparatory work: view mock poster.
- Material sent: study information, link to complete web-based survey, mock poster, and 2 scientific papers that support the development and validation of the CSRS [9,11].
- Objectives: (1) refine parameters of operationalized implementation strategies identified and (2) discuss the usability and usefulness of the strategies.
- Analysis: review notes, review audio recording to summarize perceptions and key insights, debrief with team, participants’ comments on the workshop summary.
- Outcomes: draw a list of the most salient strategies with operationalized parameters, summarize the implementation strategies, and share this summary with clinical research team that has expertise in syncope management.

**Excerpt of probe questions used in the workshop facilitation guide.**

**Workshop 1**

- Reviewing the barriers (participant feedback)
  Does this make sense?
  Does this resonate?
  Does this list look complete?
  Is there anything else you’d like to recommend?
  Are there other barriers you’d like to bring in our attention?
  What do you think?
  Can you talk out loud for me?
  What do you mean by that?
  Can you give me an example?
- Co-designing the strategies
  What are your initial reactions regarding those strategies?
  Which strategies would encourage you to use the Canadian Syncope Risk Score (CSRS)? What would be helpful for you to use it?
  What support would you need to implement the tool?
  Follow-up question: do cardiologists and internists need to be using the CSRS as a decision-making tool in order for physicians to accept it? Or is it simply that cardiologists and internists must accept and buy into the CSRS recommendations?

**Workshop 2**

- What type of educational strategy would make sense for you? (eg, educational face-to-face meeting, educational web-based video)
- What are your preferences regarding the mode of delivery of the educational strategy?
- What worked well in the past (when implementing a new rule in your practice)?
- What is the best way to build awareness of best practices across physician colleagues? (ie, WHO helps build awareness, HOW, and WHEN)
- Educational videos
  What did you like about it? What did you don’t like about it?
  What was effective or not in these videos? (eg, must be shorter, it’s comprehensive)
  What was the right length, duration?
- Prototype posters
  How useful or not are these QR codes?
  Where should we display this poster?
  In what extent do you think this poster would be effective to act as a prompt and a reminder for you to use CSRS?

**Workshop 3**

- Summary of evidence behind the recommendations
  How useful (or not) are they?
  How would you like to get access to them? Where should we make them accessible or available?
- Web-based calculator
  How did you find the web-based calculator?
  Where could you imagine yourself using the calculator?
  At what point during your workflow would you imagine using something like this?
  Where are you when that happens? Are you at a computer? Are you at a desk? Somewhere different depending on what patient you saw? Is there a consistent place?
  How would you like to get access to it?
- Local champion
  What does a local champion do?
  What is their role in supporting the use of CSRS?
- Feedback
  What type of information would you find useful?
  How do you want to receive it?

### Ethical Considerations

The qualitative and theoretical developmental work was formally reviewed by the institutional authorities at the Women’s College Hospital and was deemed exempt from a research ethics board approval.

### Recruitment

Participant recruitment was facilitated by the main developer of the CSRS (VT) through email communication with key informants at 3 ED sites in Ontario, Canada. Individuals who expressed an interest in participating contacted the project lead (GR) and were provided with a study information sheet via email. In each round, we aimed to recruit between 5 and 7 physicians who had different knowledge levels regarding the CSRS (ie, had heard about it and had basic or good knowledge). We used a combination of convenience and purposive sampling to ensure diversity in sex, hospital site (ie, urban academic and nonacademic), and primary language for clinical care (English or French) to capture various perspectives.

### Data Analysis

The workshops were audio recorded and the project lead (GR) listened to the complete recordings to partially transcribe key parts of the conversation to shed light on the barriers and facilitators of using the CSRS, as well as the strategies that would be helpful to improve its use. The project lead (GR) and the research assistant (KW) present during the workshops coproduced a preliminary summary of the findings. We performed a qualitative content data analysis according to the predefined objectives for each workshop by using a deductive and inductive process. We used deductive framework coding with broad categories by applying barriers previously identified as deductive codes as well as theory-based strategies. We inductively coded new strategies and operationalization parameters suggested by the participants throughout the conversation. The research team then reviewed the themes and discussed content changes. A summary of the findings and proposed changes was emailed to the participants after each workshop to solicit feedback; validate the emerging insights and operationalization parameters; and seek clarification, if needed (member checking) [32,33].

## Results

### Participant Characteristics

A total of 14 physicians participated across the 3 workshops and all were aware of the CSRS before participation. The participant characteristics are summarized in Table 1.

The following two themes were identified from the participants’ perspectives: (1) identifying and refining barriers and (2) identifying the intervention components and modes of delivery. The quotes supporting these findings are presented in Table 2.

**Table 1:** Participant demographics.

Characteristics			Workshop 1 (n=5)		Workshop 2 (n=4)		Workshop 3 (n=5)
Sex ratio (female:male)			2:3		1:3		2:3
Years practicing medicine, mean (SD; range)			8.4 (2.70; 4-11)		11.9 (8.53; 4-19.5)		7 (2.62; 3.5-10)
**Site (ED^a^), n (%)**							
	English academic hospital	2 (40)		2 (50)		3 (60)	
	French academic hospital	2 (40)		1 (25)		1 (20)	
	English nonacademic hospital	1 (20)		1 (25)		1 (20)	

^a^ED: emergency department.

**Table 2:** Themes supported by participants’ quotes.

Results				Quotes
**Theme 1: identifying and refining barriers**				
	Discomfort of using the CSRS^a^—feeling of hesitancy			“We reviewed this in journal club at [our hospital] and the biggest thing that came up and common to clinical decision rule is the clinical Gestalt at the end [...] Physicians felt hesitant using tool arguing it is telling me if it is vasovagal or cardiac syncope, seems counterintuitive to make decision in order to use tool to tell you something you already know.” [Workshop 2, Participant 2, Female]
	Lack of collective buy-in			“For emerg[ency] doctors you are worried to accept the responsibility of discharging people at home. I think there needs to be something coming from cardiologists to say ‘yes, that is acceptable to discharge someone home’; you need to know there is a timely follow-up; it is hard to adopt rule if we don’t feel it is accepted widely by specialists as well. So, we feel supported to safely use it.” [Workshop 1, Participant 1, Female]
**Theme 2: identifying the intervention components and modes of delivery to overcome the identified barriers**				
	**Subtheme 2.1: generating high-level strategies and developing strategies prototypes**			
		Targeting broad audience when implementing strategies	“Grand rounds, presentations during physicians’ meetings, posters, study sheets all over the place, research assistants remind you to use it; seeing publications, seeing it on social media, pretty embedded in our group; importance of multiple strategies.” [Workshop 1, Participant 4, Male]	
		Different formats for educational strategies	“I think case-based rounds is great. I think to get hospital buy-in I’m thinking smaller community hospitals. I think having combined rounds with cardiology, medicine, and emerg[ency] to go over the score and how to apply it, and what are monitoring implications—I think that’s helpful as a group. That way the discussion happens with all the key players and the barriers to implementing this. As opposed to presenting it in silos really when a patient comes all these people are important, so combining a strategy could be helpful.” [Workshop 2, Participant 2, Female]	
		Poster display at ED^b^	“Flow diagram of what you would do with each category; I think a lot of people in emergency medicine like ‘if this, then that,’ to know which way to flow. That can help to take some of the thought process out if it as long as it is standardized across colleagues/specialists. We want to be practicing along with our colleagues and specialists, so having consensus with colleagues to follow the diagram with appropriate clinical practice and applying the rule appropriately, I think would help too.” [Workshop 1, Participant 4, Male]	
		Local champion	“After the six months check-up can be within the department if you have that champion, is the one that can do that link up. The first six months I think will give you enough information, does that local champion can be the one and that links back with the research team and see what is it at that point, having someone locally I think is significantly better to get like off the cuff comments and things like that and how they wish it was changed, applied or supports, I think it has better chance of getting quality feedback and regular feedback.” [Workshop 3, Participant 3, Male]	
	**Subtheme 2.2: refining and testing strategies**			
		Poster	“I can refer to that poster, maybe give me a little bit of credibility if I’m advocating for an admission where I’m getting pushed back.” [Workshop 3, Participant 2, Male]	
		Web-based calculator	“Thinking back to other scores, or decision rules that are on calculator...It does bug me sometimes when I’m not able to access like a summary of why that’s the recommendation or why that’s the rule but again having an optional because if you already know it you don’t need to come up every time if you forget or you want to know about the medium risk what exactly are the details having the option to go easily access from the rule would be nice.” [Workshop 3, Participant 5, Female]	
		Feedback	This quote speaks to quality indicators that would be of interest: “I think for me anecdotal feedback is really helpful. So with the implementation of like the electronic records and EPIC [EMR^c^], actually getting responses from the referrals that I make and similarly like for this type of thing, getting even anecdotal [...] feedback from cardiology on the results of the Holter monitor well, over time, I think, build up to convince me to use the rule. So I think that there should be someone at each site who’s trying to collect that information, like, based on what was the risk level patient has, did they have a Holter or not? And are the numbers that were seeing, matching up what the what the actual CSRS showed.” [Workshop 3, Participant 5, Female]	

^a^CSRS: Canadian Syncope Risk Score.

^b^ED: emergency department.

^c^EMR: electronic medical record.

### Theme 1: Identifying and Refining Barriers

Workshop participants validated the following barriers identified in previous study: discomfort using the CSRS, the lack of confidence, the lack of knowledge and skills, and uncertainty around interpretation. Throughout the workshops, physicians highlighted additional barriers, including struggling with how to apply the CSRS recommendations, the inapplicability of the CSRS for some patient clinical presentations, the lack of the CSRS buy-in from the broader medical team (ie, cardiologists and internists), the lack of evidence about the effectiveness of the CSRS tool, and its practice-based recommendations on patient outcomes. The lack of collective buy-in is an important barrier to CSRS use, as physicians have described the importance of all team members. The mapping exercise of linking barriers to theoretical determinants is presented in Multimedia Appendix 1, along with examples on how BCTs (eg, credible source, pros and cons, and instruction on how to perform the behavior) can be operationalized.

### Theme 2: Identifying the Intervention Components and Modes of Delivery

#### Subtheme 2.1: Generating High-Level Strategies and Developing Strategies Prototypes

Participants described that effectively addressing the identified barriers required multiple strategies deployed using various dissemination channels. The need to target a broad audience (ie, emergency medicine physicians, cardiologists, internists, head of department, and nursing staff) with consistent exposure over time (ie, repeat messaging) was emphasized. Participants also highlighted the need to leverage existing structures, including integration of the CSRS into the electronic medical record (EMR), discussing the CSRS at standing educational meetings, and displaying a poster in the workplace environment. There was an agreement that educational meetings could be used to promote general awareness of the CSRS and to encourage a nuanced discussion about its application. However, a range of opinions were expressed on the best format for those educational meetings (eg, combined grand rounds and case-based discussions in small groups). Participants felt that holding combined grand rounds with emergency medicine physicians and specialists (ie, cardiologists and internists) would be a coordinated strategy to address multiple barriers simultaneously. How these educational events are promoted is important to stimulate interest and excitement, including highlighting the credibility of the speaker. Having a journal club with emergency medicine physicians and specialists to review evidence around the CSRS would be useful. Podcasts can be another interesting channel to disseminate knowledge around the CSRS. Participants shared the example of *Emergency Medicine Reviews and Perspectives* [34], a perceived trusted web-based resource, which is a monthly emergency medicine audio series encompassing continuing medical education. Displaying a poster in the ED was suggested as a helpful visual cue, with participants describing the usefulness of the computed tomography head rule poster [35,36] as an example. Participants also highlighted the need for a local champion that could play multiple roles to model the application and use of the CSRS, to influence uptake among colleagues, speak in educational meetings, to facilitate connection between the clinical and research teams, monitor the implementation process over time, and provide in-person or written feedback.

On the basis of these insights, the research team created 2 prototype posters (Multimedia Appendix 3) and prepared a summary of evidence. The 2 posters encompassed similar content with different displays. In poster 1, information around “For whom the CSRS must be applied” and “When to use CSRS” was highlighted. Poster 2 focused on the proposed course of treatment before the application of the CSRS. In both prototype posters, the CSRS was illustrated along with the 3 risk levels and their proposed practice-based recommendations. In response to participants’ feedback, QR codes were added to the posters: (1) how to use the CSRS, (2) recommendation evidence, and (3) web-based calculator. We prepared a brief summary of evidence to support each practice recommendation for low-risk, medium-risk, and high-risk patients with the intention that physicians feel more confident to use the CSRS and apply the subsequent practice-based recommendations.

The research team also leverages the following existing strategies: educational videos displaying evidence of how the CSRS has been developed and validated and the way to use it, web-based calculators, and email communications used to prompt physicians to use the CSRS and to provide them with positive feedback. The email communication was drawn from a previous pilot study on remote cardiac monitoring as an example to communicate positive feedback on patient impact (ie, an example where home monitoring detected a patient arrhythmia) as well as messaging to remind physicians to use the CSRS.

#### Subtheme 2.2: Refining and Testing Strategies

Participants discussed the perceived usability, usefulness, and operationalization of the following strategies: educational videos, the poster, the web-based calculator, the summary of evidence, the local champion, and the email communication.

Participants found that the components and features (eg, written summaries, questions, graphics, videos, and links to scientific papers) of educational videos were perceived as useful, and the content was perceived as clear, concise, relevant, and credible. The duration of videos was reasonable if viewed out of the workplace but was too long to be viewed during a shift. It was suggested that a 5-minute video that includes the main information would be an ideal length and would facilitate wider dissemination of the CSRS.

When reviewing the 2 poster prototypes, participants suggested the need to simplify the posters, separating the explicative notes (ie, additional information) from the care pathway, and move those notes as footnotes using a different font (eg, smaller fonts for footnotes) to make the content easier to read. They found poster 2 usable, that is, easy to follow, simple, and appealing. They would use it as a reminder and as a prompt to apply the CSRS, which would be helpful especially at the early stages of the CSRS implementation process in the ED. They would also refer their colleagues to this poster, which is seen as a way of giving credibility to their ED syncope management course of treatment. However, participants identified the following barriers to using such a poster: the risk of poster fatigue, lack of space to display it in their clinical settings, and lack of skills in using QR codes. They suggested to keep only one QR code in the poster, that is, the one related to the web-based calculator.

All participants tested the CSRS web-based calculator [37]. They suggested ways of improving the usability of the web-based calculator: (1) reviewing the wording of some criteria to avoid misleading interpretations, (2) adding a “not drawn” response option to this question “elevated troponin level,” (3) adding access to evidence, and (4) adding access to practice-based recommendations for low-, medium-, and high-risk patients (ie, what to do with the risk score). All participants intended to use the web-based calculator but for different purposes: use in practice and as an educational tool for medical students. Some would use it only if it is integrated into the EMR and will not use it if it is part of a mobile app. Participants would find it useful to discuss in length the summary of evidence in grand rounds or in another type of educational meeting as an initial evidence uptake. Obtaining easy access to evidence was considered important; tying evidence to a web-based calculator and to the EMR would be one way to improve its access.

Finally, email communication with feedback would be useful for emergency medicine physicians to convince them to use the CSRS. Participants had different opinions on how and by whom feedback could be delivered, such as through educational outreach, one-to-one discussion with local champion, and email communication. They would like to receive feedback from the research team (especially the CSRS developers) and a cardiologist within their hospital.

## Discussion

### Principal Findings

The parameters of these strategies are outlined in Table 3. These strategies will be further developed and deployed as part of a nested process evaluation for a stepped wedge cluster trial.

This work was built on a comprehensive and systematic intervention development process anchored in 1 previous qualitative study and in theoretical mapping of linking theoretical determinants with evidence-based strategies. Furthermore, the contribution of this work is to have involved physicians at different stages to gain insight about the perceived barriers and to test strategies in their context. Key barriers included uncertainty about when and how to apply the CSRS recommendations, the lack of resources (eg, cardiac monitors), the lack of buy-in from the broader medical team, discomfort (hesitancy) using the CSRS, and the lack of evidence about the impact on patient outcomes. Surprisingly, no reference on workload or time constraint was brought up, as is often found in other studies [38-41]. Our findings suggest that physician capability should be a central target of implementation supports, specifically the capability to interpret CSRS-based criteria and apply them across a range of clinical presentations.

**Table 3:** Summary of the strategies and their parameters over the 3 workshops.

Strategy	Required content	Mode of delivery	Delivery source	Target audience	Target outcome
Educational meetings (eg, grand rounds) and videos	Nuances, barriers, and pitfalls when using the CSRS^a^; evidence underlying CSRS and recommendations; cost and resources; how to deal with ultra–low-risk criteria and troponin; what to do with risk score	Web-based In-person	CSRS experts, cardiology, and general medicine physicians	All locations where CSRS will be applied Diverse stakeholders^b^	Improve knowledge of and comfort in using the CSRS. Improve skills on how to use the CSRS.
Web-based calculator	How to deal with troponin criterion; what to do with the risk score	Web^c^, mobile app, and EMR^d^	Electronic content	CSRS users^e^	Improve CSRS integration into workflow
CSRS integration into the EMR	Interpretation of the risk score; what to do with the risk score	EMR	Electronic content	CSRS users^e^	Improve CSRS integration into workflow
Local champion	Roles: speaker, monitor the implementation process, adapt strategies; provide support and feedback. Attributes: strong and positive leadership skills, know how to apply CSRS and recommendations.	In-person	Local emergency medicine physicians and cardiologists (each site)	CSRS users^e^	Improve collective buy-in
Poster	Care pathway, how to deal with troponin criterion	Paper QR codes	Paper-based	Diverse stakeholders^b^	Improve collective buy-in
Dissemination of evidence summary	Impact of CSRS practice-based recommendations on patient outcomes. Research papers—CSRS development and validation	On the internet In-person Journal clubs	Electronic content CSRS experts	Diverse stakeholders^b^	Improve knowledge
Feedback	CSRS impacts on providers’ practice; numbers of cardiac monitor referrals and of arrythmias detected	In-person and written	Champions	CSRS users^e^	Improve skills and adoption of behavior (CSRS uptake)
Prompts	Invitation to use CSRS, image with arrythmia detected (feedback)	Email communication	CSRS experts Champions	CSRS users^e^	Improve social opportunity

^a^CSRS: Canadian Syncope Risk Score.

^b^Emergency medicine physicians, family physicians working at emergency department, any consultants who are asked for high-risk patients, cardiologists, internists, nurses (including nurse practitioners), and support from head of department.

^c^MDCalc [37].

^d^EMR: electronic medical record.

^e^All emergency medicine physicians and residents.

### Comparison With Prior Work

Training is an evidence-based and frequently used strategy to build physician capability by increasing their skills [23]. This can be operationalized through a variety of mechanisms, including seminars, interactive workshops, and teaching programs such as simulation and training sessions [42,43]. In our study, participants largely referred to educational meetings (eg, combined grand rounds inclusive of all relevant specialties) and educational videos. Skill building can be supplemented by creating increased opportunities to use the CSRS, including integration into the EMR, engaging local champions, displaying posters, and sending email communications to encourage use [40,41,44]. This suite of strategies is commonly used in the ED setting [44] and has demonstrated effectiveness in promoting guideline-adherent care [45].

Achieving collective buy-in across multiple specialty groups (ie, emergency medicine, cardiology, and internal medicine) was highlighted as an essential condition for successful uptake of the CSRS. Although the importance of this broader support is well documented [41,46], including in the area of risk stratification [41], the importance of designing implementation strategies targeting this broader audience (ie, an audience beyond the immediate end user) has been unexplored. Although ED syncope care primarily rests on the shoulders of the emergency physician, support from experts in cardiology, internal medicine, and hospitalists is needed for care of those with suspected or identified serious conditions and further inpatient or outpatient investigations. Physicians rely on their colleagues and professional networks as a unique source of tacit knowledge that serve to either validate initial reasoning or offer alternative approaches [47]. This presents an opportunity to influence uptake through existing channels of social influence that extend beyond the primary setting of interest (in this case, the ED). Strategies may benefit from alignment with the underlying factors that influence patterns of collaboration, including perceived reputational value, experiential information (including personal relationships and visibility), professional identity, and self-awareness of competence [48]. In addition, strategies that target components such as champion or opinion leader, social support, and credible source would be promising ingredients to consider [27].

Similar to the study by Bravo et al [49], our findings highlight a tension between user preferences and scientific evidence and the critical role of triangulating user input in addition to scientific evidence to leverage both sources of knowledge in the design of implementation strategies. Specifically, physician participants identified education in the form of grand rounds as a strategy to address areas of uncertainty and to improve collective awareness of the tool within the interdisciplinary team. Although education effectively increases knowledge and awareness, it is training that effectively builds or strengthens skills [23]. On the basis of physicians’ perceived barrier of not knowing how to apply CSRS, education alone might not be sufficient because it has to do with developing abilities to apply the tool among various patients. In such cases, training would be more suitable. Simply put, each strategy has its own function and mechanisms, allowing it to overcome barriers to using the CSRS and strengthen the facilitators.

### Limitations

We used a combination of purposive and convenience sampling to recruit emergency medicine physicians working at 3 different hospitals; therefore, the results may not reflect the experiences of physicians working at other sites. Furthermore, all participants were aware of the CSRS before their participation in the workshops, with most being employed at the same hospital where the CSRS has been piloted. Future studies should explore whether the resulting strategies align with and effectively address the barriers experienced by those who are unaware of the CSRS. Finally, although a comprehensive list of potential BCTs was developed (Multimedia Appendix 1), only a subset of these were prioritized for discussion in the workshops because of feasibility and time constraints. A more comprehensive discussion would have yielded additional strategies to address the identified barriers, and future work should assess whether barriers persist that might be amenable to strategies that were not thoroughly considered. For example, we could operationalize the identification and the preparation of local champions more extensively, and we could target which skills would need to be addressed in a training and how we should impart them (eg, simulation and small-group workshop with demonstration on how to use the CSRS with different patients’ clinical presentations). The provision of performance feedback on the accurate use of the CSRS for risk-stratifying patients by experts (eg, cardiologists and CSRS developers) could also be an avenue to consider.

### Conclusions

The ability of the CSRS to effectively improve patient safety and ED syncope management relies on broad buy-in and uptake by physicians. To ensure that the CSRS is well positioned for impact, we identified and developed a comprehensive suite of implementation strategies, including posters, educational meetings (grand rounds), educational videos (with a training component on how to apply the CSRS among various patients), the integration of the CSRS into the EMR, and a web-based calculator to calculate the risk score. These strategies will be evaluated to understand whether and how they are being implemented in practice and whether they are effective in addressing the identified barriers with the objective of improving syncope management in EDs. The next phase of work involves an embedded process evaluation that will provide insight into whether and how this UCD systematic development approach facilitates the ability to effectively target preidentified barriers, physician engagement with the implementation strategies, and broader uptake of the CSRS.

## Appendix

Multimedia Appendix 1 Excerpt of the process of identifying the intervention components and modes of delivery to overcome the identified barriers.

Multimedia Appendix 2 COREQ (Consolidated Criteria for Reporting Qualitative Studies) checklist.

Multimedia Appendix 3 Prototype posters.

## Abbreviations

BCT: behavior change technique
COM-B: Capability, Opportunity, Motivation, and Behavior
COREQ: Consolidated Criteria for Reporting Qualitative Studies
CSRS: Canadian Syncope Risk Score
ED: emergency department
EMR: electronic medical record
TDF: Theoretical Domains Framework
UCD: user-centered design

## References

[1] Brignole M, Moya A, de Lange FJ, Deharo JC, Elliott PM, Fanciulli A, Fedorowski A, Furlan R, Kenny RA, Martín A, Probst V, Reed MJ, Rice CP, Sutton R, Ungar A, van Dijk JG, ESC Scientific Document Group (2018). 2018 ESC guidelines for the diagnosis and management of syncope. Eur Heart J.

[2] Brignole M, Menozzi C, Bartoletti A, Giada F, Lagi A, Ungar A, Ponassi I, Mussi C, Maggi R, Re G, Furlan R, Rovelli G, Ponzi P, Scivales A (2006). A new management of syncope: prospective systematic guideline-based evaluation of patients referred urgently to general hospitals. Eur Heart J.

[3] Sandhu RK, Sheldon RS (2019). Syncope in the emergency department. Front Cardiovasc Med.

[4] Soteriades ES, Evans JC, Larson MG, Chen MH, Chen L, Benjamin EJ, Levy D (2002). Incidence and prognosis of syncope. N Engl J Med.

[5] Almulhim KN (2022). The characteristics of syncope-related emergency department visits: resource utilization and admission rate patterns in emergency departments. Cureus.

[6] Thiruganasambandamoorthy V, Hess EP, Turko E, Perry JJ, Wells GA, Stiell IG (2013). Outcomes in Canadian emergency department syncope patients--are we doing a good job?. J Emerg Med.

[7] Sandhu RK, Tran DT, Sheldon RS, Kaul P (2018). A population-based cohort study evaluating outcomes and costs for syncope presentations to the emergency department. JACC Clin Electrophysiol.

[8] Solbiati M, Bozzano V, Barbic F, Casazza G, Dipaola F, Quinn JV, Reed MJ, Sheldon RS, Shen WK, Sun BC, Thiruganasambandamoorthy V, Furlan R, Costantino G (2018). Outcomes in syncope research: a systematic review and critical appraisal. Intern Emerg Med.

[9] Thiruganasambandamoorthy V, Kwong K, Wells GA, Sivilotti ML, Mukarram M, Rowe BH, Lang E, Perry JJ, Sheldon R, Stiell IG, Taljaard M (2016). Development of the Canadian syncope risk score to predict serious adverse events after emergency department assessment of syncope. CMAJ.

[10] Shen W, Sheldon RS, Benditt DG, Cohen MI, Forman DE, Goldberger ZD, Grubb BP, Hamdan MH, Krahn AD, Link MS, Olshansky B, Raj SR, Sandhu RK, Sorajja D, Sun BC, Yancy CW (2017). 2017 ACC/AHA/HRS guideline for the evaluation and management of patients with syncope: executive summary: a report of the American college of cardiology/American heart association task force on clinical practice guidelines and the heart rhythm society. J Am Coll Cardiol.

[11] Thiruganasambandamoorthy V, Sivilotti ML, Le Sage N, Yan JW, Huang P, Hegdekar M, Mercier E, Mukarram M, Nemnom MJ, McRae AD, Rowe BH, Stiell IG, Wells GA, Krahn AD, Taljaard M (2020). Multicenter emergency department validation of the Canadian syncope risk score. JAMA Intern Med.

[12] Curran GM (2020). Implementation science made too simple: a teaching tool. Implement Sci Commun.

[13] Damschroder LJ, Aron DC, Keith RE, Kirsh SR, Alexander JA, Lowery JC (2009). Fostering implementation of health services research findings into practice: a consolidated framework for advancing implementation science. Implement Sci.

[14] Squires JE, Aloisio LD, Grimshaw JM, Bashir K, Dorrance K, Coughlin M, Hutchinson AM, Francis J, Michie S, Sales A, Brehaut J, Curran J, Ivers N, Lavis J, Noseworthy T, Vine J, Hillmer M, Graham ID (2019). Attributes of context relevant to healthcare professionals' use of research evidence in clinical practice: a multi-study analysis. Implement Sci.

[15] Marshall M, de Silva D, Cruickshank L, Shand J, Wei L, Anderson J (2017). What we know about designing an effective improvement intervention (but too often fail to put into practice). BMJ Qual Saf.

[16] Desveaux L, Saragosa M, Russell K, McCleary N, Presseau J, Witteman HO, Schwalm JD, Ivers NM (2020). How and why a multifaceted intervention to improve adherence post-MI worked for some (and could work better for others): an outcome-driven qualitative process evaluation. BMJ Open.

[17] Dopp AR, Parisi KE, Munson SA, Lyon AR (2019). A glossary of user-centered design strategies for implementation experts. Transl Behav Med.

[18] Dopp AR, Parisi KE, Munson SA, Lyon AR (2019). Integrating implementation and user-centred design strategies to enhance the impact of health services: protocol from a concept mapping study. Health Res Policy Syst.

[19] Lyon AR, Koerner K (2016). User-centered design for psychosocial intervention development and implementation. Clin Psychol (New York).

[20] Proctor E, Silmere H, Raghavan R, Hovmand P, Aarons G, Bunger A, Griffey R, Hensley M (2011). Outcomes for implementation research: conceptual distinctions, measurement challenges, and research agenda. Adm Policy Ment Health.

[21] French SD, Green SE, O'Connor DA, McKenzie JE, Francis JJ, Michie S, Buchbinder R, Schattner P, Spike N, Grimshaw JM (2012). Developing theory-informed behaviour change interventions to implement evidence into practice: a systematic approach using the theoretical domains framework. Implement Sci.

[22] Hudek N, Brehaut JC, Rowe BH, Nguyen PA, Ghaedi B, Ishimwe AC, Fabian C, Yan JW, Sivilotti ML, Ohle R, Le Sage N, Mercier E, Archambault PM, Plourde M, Davis P, McRae AD, Hegdekar M, Thiruganasambandamoorthy V (2023). Development of practice recommendations based on the Canadian syncope risk score and identification of barriers and facilitators for implementation. CJEM.

[23] Michie S, van Stralen MM, West R (2011). The behaviour change wheel: a new method for characterising and designing behaviour change interventions. Implement Sci.

[24] Atkins L, Francis J, Islam R, O'Connor D, Patey A, Ivers N, Foy R, Duncan EM, Colquhoun H, Grimshaw JM, Lawton R, Michie S (2017). A guide to using the theoretical domains framework of behaviour change to investigate implementation problems. Implement Sci.

[25] Carey RN, Connell LE, Johnston M, Rothman AJ, de Bruin M, Kelly MP, Michie S (2019). Behavior change techniques and their mechanisms of action: a synthesis of links described in published intervention literature. Ann Behav Med.

[26] Michie S, Johnston M, Francis J, Hardeman W, Eccles M (2008). From theory to intervention: mapping theoretically derived behavioural determinants to behaviour change techniques. Appl Psychol.

[27] Michie S, Richardson M, Johnston M, Abraham C, Francis J, Hardeman W, Eccles MP, Cane J, Wood CE (2013). The behavior change technique taxonomy (v1) of 93 hierarchically clustered techniques: building an international consensus for the reporting of behavior change interventions. Ann Behav Med.

[28] D'Lima D, Lorencatto F, Michie S, Nilsen P, Birken SA (2020). The behaviour change wheel approach. Handbook on Implementation Science.

[29] Crane D (2013). Behaviour change technique taxonomy v1 (BCTTv1). Apple Inc.

[30] The theory and techniques tool. Human Behaviour-Change Project.

[31] Tong A, Sainsbury P, Craig J (2007). Consolidated criteria for reporting qualitative research (COREQ): a 32-item checklist for interviews and focus groups. Int J Qual Health Care.

[32] Cypress BS (2017). Rigor or reliability and validity in qualitative research: perspectives, strategies, reconceptualization, and recommendations. Dimens Crit Care Nurs.

[33] Lincoln YS, Guba EG (1985). Naturalistic Inquiry.

[34] EM: RAP | Emergency medicine reviews and perspectives.

[35] Lewis D (2014). New SJRHEM guideline – minor head injury. Emergency Medicine.

[36] Stiell IG, Wells GA, Vandemheen K, Clement C, Lesiuk H, Laupacis A, McKnight RD, Verbeek R, Brison R, Cass D, Eisenhauer ME, Greenberg G, Worthington J (2001). The Canadian CT head rule for patients with minor head injury. Lancet.

[37] Thiruganasambandamoorthy V (2021). Canadian syncope risk score. MDCalc.

[38] Kingston M, Griffiths R, Hutchings H, Porter A, Russell I, Snooks H (2020). Emergency admission risk stratification tools in UK primary care: a cross-sectional survey of availability and use. Br J Gen Pract.

[39] Muthee TB, Kimathi D, Richards GC, Etyang A, Nunan D, Williams V, Heneghan C (2020). Factors influencing the implementation of cardiovascular risk scoring in primary care: a mixed-method systematic review. Implement Sci.

[40] Wilson M, Keeley J, Kingman M, Wang J, Rogers F (2020). Current clinical utilization of risk assessment tools in pulmonary arterial hypertension: a descriptive survey of facilitation strategies, patterns, and barriers to use in the United States. Pulm Circ.

[41] Westafer LM, Kunz A, Bugajska P, Hughes A, Mazor KM, Schoenfeld EM, Stefan MS, Lindenauer PK (2020). Provider perspectives on the use of evidence-based risk stratification tools in the evaluation of pulmonary embolism: a qualitative study. Acad Emerg Med.

[42] Gamage US, Mahesh PK, Schnall J, Mikkelsen L, Hart JD, Chowdhury H, Li H, McLaughlin D, Lopez AD (2020). Effectiveness of training interventions to improve quality of medical certification of cause of death: systematic review and meta-analysis. BMC Med.

[43] Lateef F (2010). Simulation-based learning: just like the real thing. J Emerg Trauma Shock.

[44] de Wit K, Curran J, Thoma B, Dowling S, Lang E, Kuljic N, Perry JJ, Morrison L (2018). Review of implementation strategies to change healthcare provider behaviour in the emergency department. CJEM.

[45] Ebben RH, Siqeca F, Madsen UR, Vloet LC, van Achterberg T (2018). Effectiveness of implementation strategies for the improvement of guideline and protocol adherence in emergency care: a systematic review. BMJ Open.

[46] Dmitriew C, Ohle R (2021). Barriers and facilitators affecting implementation of the Canadian clinical practice guidelines for the diagnosis of acute aortic syndrome. Implement Sci Commun.

[47] Cohen DA, Levy M, Cohen Castel O, Karkabi K (2013). The influence of a professional physician network on clinical decision making. Patient Educ Couns.

[48] Kierkegaard P, Owen-Smith J (2021). Determinants of physician networks: an ethnographic study examining the processes that inform patterns of collaboration and referral decision-making among physicians. BMJ Open.

[49] Bravo CA, Llovet D, Witteman HO, Desveaux L, Presseau J, Saragosa M, Vaisson G, Umar S, Tinmouth J, Ivers NM (2018). Designing emails aimed at increasing family physicians' use of a web-based audit and feedback tool to improve cancer screening rates: cocreation process. JMIR Hum Factors.

