# Supplementary material for: Developing Implementation Strategies to Support the Uptake of a Risk Tool to Aid Physicians in the Clinical Management of Patients With Syncope: Systematic Theoretical and User-Centered Design Approach
Source: JMIR Hum Factors. 2023 Jun 13;10:e44089. doi: 10.2196/44089 (PMC10337431; doi:10.2196/44089)
Supplement: Multimedia Appendix 1 [file humanfactors_v10i1e44089_app1.pdf]

Multimedia Appendix 1. Excerpt of the process of identifying the intervention components and modes of delivery to overcome the identified barriers

**Mapping the barriers to TDF and COM-B**		**Identifying the intervention components and modes of delivery to overcome the identified barriers [23, 25-28, 47,48]**		
**Which barriers of using CSRS need to be addressed?**	**Within which theoretical domains do the barriers operate?**	**Potential BCT [25,26, 47,48]**	**Definition and Description – How we could operationalize this**	**Desired outcomes or change**
Physician discomfort in using CSRS	TDF: Social/professional role & Identity<br><br>COM-B: Motivation Reflective	✓Social support<br>✓Credible source<br>*IF*: Education, Persuasion, Modelling	Identify and prepare champion<br>✓ *Example:* Present a speech given by a renown and trusted MD in ED (high status professional/credible source such as cardiologist) to emphasize the reliability of CSRS and how it can be implemented within teamwork.	Enhance confidence in using the CSRS tool
Unresolved question about included/ excluded criteria, weighting, or aspects of recommendations of CSRS	TDF: Knowledge<br><br>COM-B: Capability Psychological	✓Information about health and social consequences<br>✓Instruction on how to perform the behaviour<br>✓Feedback on behaviour<br>✓*IF*: Education, training	Instruction on how to perform behaviour<br>✓ *Example:* Providing information on the whole decision-making process.<br>*Example:* Providing training (cognitive/skills) on how to apply CSRS tool	Increase knowledge about the CSRS<br><br>Boost self-efficacy
Lack of confidence in CSRS / validity evidence	TDF: Beliefs about consequences	✓Information about health consequences<br>✓Information about social and environmental consequences<br>✓Pros and cons	Pros and Cons<br>✓ *Example:* List and compare advantages (e.g., complete high-risk investigations more quickly) and disadvantages of using CSRS	Increase physicians' knowledge on 30-Day Serious Outcomes after ED disposition according to low, medium and high-risk patients
Physician confusion/ lack of knowledge regarding criteria or recommendations	TDF: Knowledge<br><br>COM-B: Capability/Psychological	✓Information regarding behaviour, outcome	Information regarding behaviour<br>✓ *Example:* Provide physicians' knowledge about eligibility criteria of when using the CSRS and not using it, for whom, in what circumstances	Increase physician knowledge of inclusion and exclusion criteria, how to evaluate the criteria and how interpret the score

Legends: BCT: Behaviour change techniques; COM-B: Capability, Opportunity, Motivation, and Behaviour; ED: emergency departments; IF: Intervention function; MD: Medical doctor; TDF: Theoretical Domains Framework.
