# Supplementary material for: Developing Implementation Strategies to Support the Uptake of a Risk Tool to Aid Physicians in the Clinical Management of Patients With Syncope: Systematic Theoretical and User-Centered Design Approach
Source: JMIR Hum Factors. 2023 Jun 13;10:e44089. doi: 10.2196/44089 (PMC10337431; doi:10.2196/44089)
Supplement: Multimedia Appendix 2 [file humanfactors_v10i1e44089_app2.pdf]

Multimedia Appendix 2. COREQ: Consolidated Criteria for Reporting Qualitative Studies

Item No.	Topic	Qualitative description	Reported on page no./Section of the paper
**Domain 1: Research team and reflexivity**			
Personal characteristics			
1.	Interviewer/facilitator	The postdoctoral researcher, GR	
2.	Credentials	GR – Postdoctoral researcher –PhD, RN (Nursing)	Title page
		VT – MBBS, CCFP-EM, M.Sc	
		KW – M.Sc	
		BG – M.Sc	
		PAN – B.Sc	
		LD– PhD, PT	
3.	Occupation	GR –Postdoctoral researcher, Women's College Hospital, Toronto, Canada	Not reported in the main text
		VT - Professor, Department of Emergency Medicine; School of Epidemiology & Public Health, University of Ottawa; Senior Scientist, Clinical Epidemiology Program, Ottawa Hospital Research Institute in Ottawa, Canada.	
		KW – Research assistant II, Institute for Health System Solutions and Virtual Care, Women's College Hospital	
		BG - Clinical Research Coordinator, The Ottawa Hospital	
		PAN - Clinical Research Assistant II, The Ottawa Hospital	
		LD - Scientific Lead, Trillium Health Partner's Institute for Better Health; Innovation Fellow, Women's College Hospital Institute for Health System Solutions and Virtual Care; Assistant	

		Professor, Institute of Health Policy, Management and Evaluation, University of Toronto, Canada	
4.	Gender	The research team is composed of five female-identifying and one-male identifying (interviewer: female)	Title page
5.	Experience and training	GR –has experience in conducting qualitative research, including conducting online data collection such as focus groups, and in developing interventions to build patients and providers' capabilities.	Not reported
		VT– has clinical and research-based expertise in syncope care and in developing and validating risk stratification tool (CSRS).	
		KW – is developing skillsets in implementation science and in qualitative research.	
		BG – has been involved in previous works surrounding the development and validation of the CSRS.	
		PAN – has been involved in previous works surrounding the development and validation of the CSRS.	
		LD– has expertise in implementation science/behavioural science, mixed methods methodologies, knowledge-translation, evidence-based decision-making process, and co-design approaches.	
Relationship with participants			
6.	Relationship established	GR encouraged participants to express themselves freely, by soliciting their feedback and to get their inputs in co-designing implementation strategies.	Methods section
7.	Participant knowledge of interviewer	GR introduced herself to participants as a postdoctoral researcher who leads the co-development process of implementation strategies and not as a clinical content expert.	Not reported in the main text

8.	Interviewer characteristics	GR is a postdoctoral researcher, supervised by LD.	Not reported
**Domain 2: Study design**			
Theoretical framework			
9.	Methodological orientation and theory	Described in Step 3: Identifying the intervention components and modes of delivery to overcome the identified barriers	Methods section
Participant selection			
10.	Sampling	Described in recruitment section	Methods section
11.	Method of approach	Described in recruitment section	Methods section
12.	Sample size	Described in the Participant characteristics section	Results section
13.	Non-participation	N/A	
Setting			
14.	Setting of data collection	Described in Step 3: Identifying the intervention components and modes of delivery to overcome the identified barriers	Methods section
15.	Presence of non-participants	N/A	
16.	Description of sample	See Table 3: Participant demographics	Results section
Data collection			
17.	Interview guide	See Table 2 – Excerpt of probe questions used in the workshop facilitation guide	Methods section
18.	Repeat workshops	No	
19.	Audio/visual recording	Described in Data analysis	Methods section
20.	Field notes	See Table 1 - Workshops -related processes and content	Methods section

p.21.	Duration	Described in Step 3: Identifying the intervention components and modes of delivery to overcome the identified barriers	Methods section
22.	Data saturation	Described in Step 3: Identifying the intervention components and modes of delivery to overcome the identified barriers	Methods section
23.	Transcripts returned	Described in Data analysis section.	Methods section
**Domain 3: Analysis and findings**			
Data analysis			
24.	Number of data coders	Described in Data analysis section.	Methods section
25.	Description of the coding tree	We didn't describe the coding tree, but we clearly provided a narrative to describe the content of each theme.	Not reported
26.	Derivation of themes	Described in Data analysis section.	Methods section
27.	Software	Not used	Not reported
28.	Participant checking	Participants received a summary of the workshop they were involved in.<br>Described in Data analysis section.	Methods section
Reporting			
29.	Quotations presented	See Table 4. Themes supported by participants' quotes.	Results section
30.	Data and findings consistent	The findings are strongly supported by the qualitative data.	Results section
31.	Clarity of major themes	Themes 1 and 2	Results section
