# Supplementary material for: Developing Implementation Strategies to Support the Uptake of a Risk Tool to Aid Physicians in the Clinical Management of Patients With Syncope: Systematic Theoretical and User-Centered Design Approach
Source: JMIR Hum Factors. 2023 Jun 13;10:e44089. doi: 10.2196/44089 (PMC10337431; doi:10.2196/44089)
Supplement: Multimedia Appendix 3 [file humanfactors_v10i1e44089_app3.docx]

## Multimedia Appendix 3. Prototype posters

**Poster #1 – Workshop #2**

**Canadian Syncope Risk Score based Practice Recommendations**

- For use among ED patients who present within 24-hours of their syncope^1^
- After initial ED assessment (history, physical exam ± orthostatic vitals and ECG)^2^ if a serious condition is suspected investigate accordingly
- Does not apply to patients who had a serious condition identified in the ED or those who require consultation/hospitalization not related to syncope work-up
- Patients who fulfill ultra-low-risk criteria^3^ do not require troponin measured and can be assumed as normal

| **CANADIAN SYNCOPE RISK SCORE** | |
| --- | --- |
| **Category** | **Points** |
| **Clinical evaluation** |  |
| Predisposition to vasovagal symptoms^4^ | -1 |
| History of heart disease^5^ | 1 |
| Any systolic pressure reading <90 or >180 mm Hg^6^ | 2 |
| **Investigations** |  |
| Elevated troponin (>99^th^ percentile); assume as normal if patient is ultra-low-risk | 2 |
| Abnormal QRS axis (<-30 or >100^o^) | 1 |
| QRS duration >130 milliseconds | 1 |
| Corrected QT interval >480 milliseconds | 2 |
| **Diagnosis in emergency department** |  |
| Vasovagal syncope | -2 |
| Cardiac syncope | 2 |
| Cause unknown | 0 |
| **Total score** | **(-3 to 11)** |


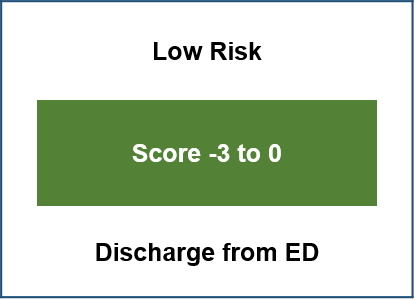

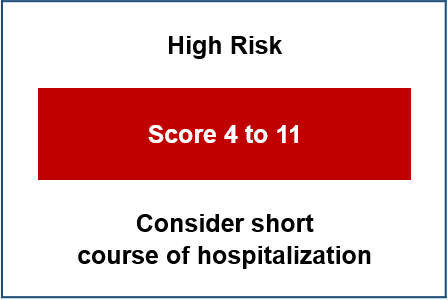

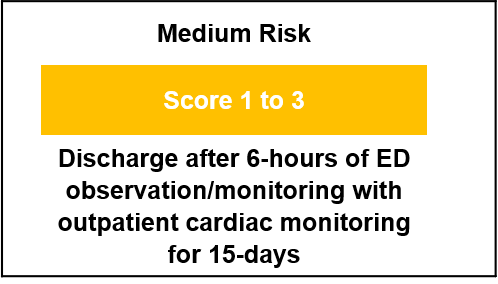


^1^Confirm the patient suffered true syncope (sudden transient loss of consciousness with spontaneous complete recovery)

^2^Routine blood tests (CBC, Chemistry) are generally not useful; pulmonary embolism prevalence is very low 0.6% investigate only if suspected; CT head is not recommended unless focal neurological deficits or head injury with risk factors as per published criteria (e.g., Canadian CT Head Rule)

^3^**Ultra-low-risk criteria:**

- - Age ≤ 50 years
  - No heart disease
  - Normal ECG (QRS axis ≥-30° to ≤+100°, QRS ≤130ms, cQT ≤480ms)
  - Clinical impression: non-cardiac syncope

^4^Triggered by being in a warm crowded place, prolonged standing, fear, emotion or pain

^5^Includes coronary or valvular heart disease, cardiomyopathy, congestive heart failure and history of non-sinus rhythm

^6^Includes blood pressure values from triage until disposition from the emergency department.

**Poster #2 – Workshop #2**


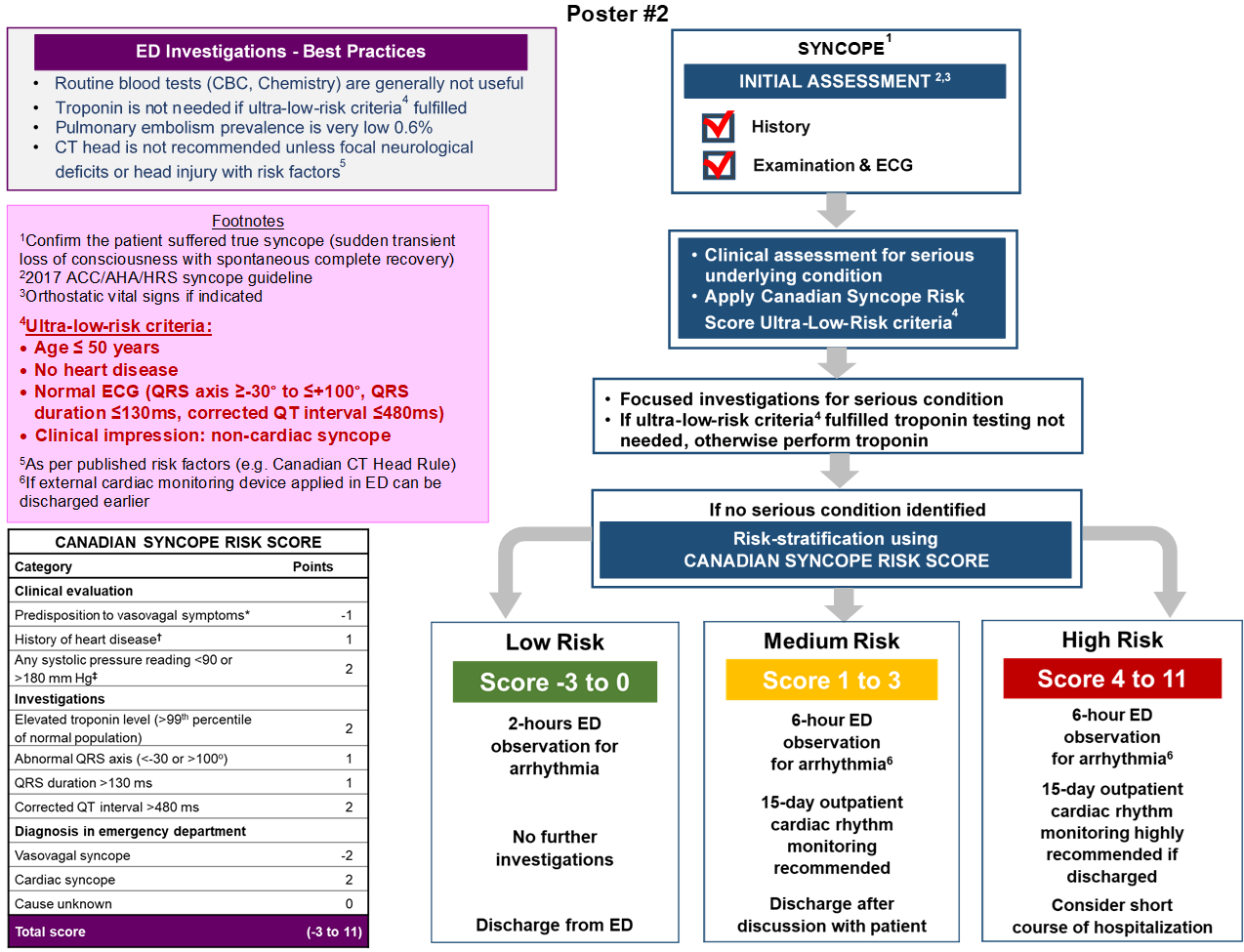


**Poster #3 – Workshop #3**

**
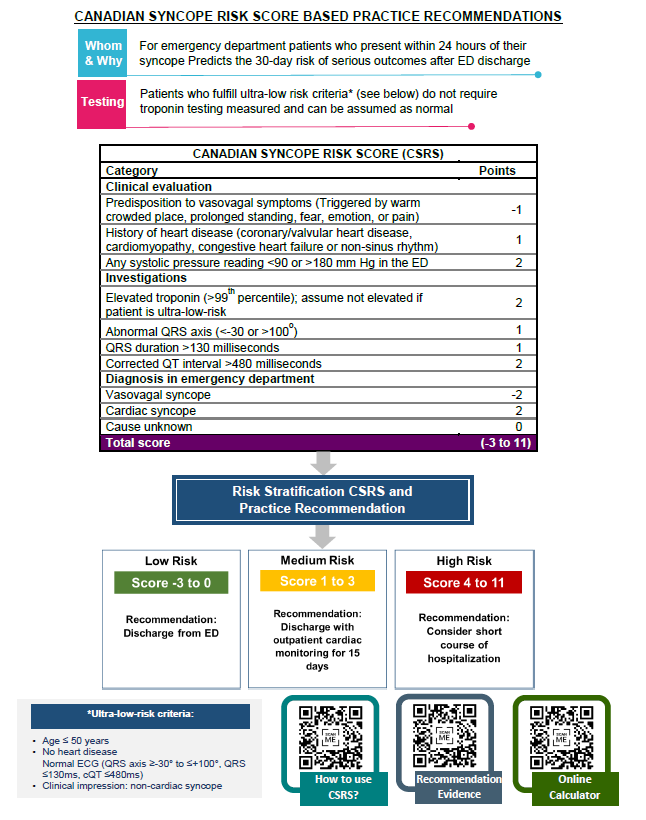
**
